# Improved Hydrogenation Kinetics of TiMn_1.52_ Alloy Coated with Palladium through Electroless Deposition

**DOI:** 10.3390/ma14081833

**Published:** 2021-04-07

**Authors:** Thabang R. Somo, Moegamat W. Davids, Mykhaylo V. Lototskyy, Mpitloane J. Hato, Kwena D. Modibane

**Affiliations:** 1 Department of Chemistry, School of Physical and Mineral Sciences, University of Limpopo (Turfloop), Polokwane, Sovenga 0727, South Africa; somoronny@gmail.com (T.R.S.); mpitloane.hato@ul.ac.za (M.J.H.); 2 HySA System, South African Institute for Advanced Material Chemistry, University of the Western Cape, Bellville, Cape Town 7535, South Africa; mwdavids@uwc.ac.za

**Keywords:** hydrogen storage materials, AB_2_ alloys, TiMn_1.52_ alloy, palladium deposition

## Abstract

The deterioration of hydrogen charging performances resulting from the surface chemical action of electrophilic gases such as CO_2_ is one of the prevailing drawbacks of TiMn_1.52_ materials. In this study, we report the effect of autocatalytic Pd deposition on the morphology, structure, and hydrogenation kinetics of TiMn_1.52_ alloy. Both the uncoated and Pd-coated materials were characterized using scanning electron microscopy/energy dispersive spectroscopy (SEM/EDS) and X-ray diffraction (XRD). XRD analyses indicated that TiMn_1.52_ alloy contains C14-type Laves phase without any second phase, while the SEM images, together with a particle size distribution histogram, showed a smooth non-porous surface with irregular-shaped particles ranging in size from 1 to 8 µm. The XRD pattern of Pd-coated alloy revealed that C14-type Laves phase was still maintained upon Pd deposition. This was further supported by calculated crystallite size of 29 nm for both materials. Furthermore, a Sieverts-type apparatus was used to study the kinetics of the alloys after pre-exposure to air and upon vacuum heating at 300 °C. The Pd-coated AB_2_ alloy exhibited good coating quality as confirmed by EDS with enhanced hydrogen absorption kinetics, even without activation. This is attributed to improved surface tolerance and a hydrogen spillover mechanism, facilitated by Pd nanoparticles. Vacuum heating at 300 °C resulted in removal of surface barriers and showed improved hydrogen absorption performances for both coated and uncoated alloys.

## 1. Introduction

AB_2_-type Laves phase alloys are an attractive class of metal hydrides due to their better reversible absorption and desorption of hydrogen, good activation property, and low cost [1,2]. The most studied and promising AB_2_-type alloy materials are the Ti–Mn binary alloys [3,4,5]. Because of their light weight, Ti–Mn binary alloys possess a large hydrogen absorption capacity of more than 1.0 hydrogen to metal ratio (H/M) and moderate equilibrium plateau pressure (reported to be 0.7 MPa) under near ambient temperatures as compared to other AB_2_ alloys [6]. Regardless of these superior properties, deterioration of hydrogen charging performances resulting from the surface chemical action of poisonous electrophilic gases is still a concern and therefore activation prior to hydrogen absorption is required [1,7]. Some well-known attempts to improve hydrogenation behaviour of these binary alloys include element substitution, structural change, and multicomponent strategies [6,8]. An example of element substitution includes a study by Liu et al. [1], where Ti and Zr comprised the A site, while Mn, Cr, V, Ni, Fe, and Cu metals occupied the B site to produce a (Ti_0.85_Zr_0.15_)_1.05_Mn_1.2_Cr_0.6_V_0.1_M_0.1_ alloy (where M=Ni, Fe, Cu). This material showed a great improvement in cyclability but poor hydrogenation kinetics due to its poor poisoning-resistance. Other improvements of the hydrogenation behaviour have been previously reported through surface protecting techniques such as microencapsulation [9,10], coating with metal oxides [11], and fluorination treatment [12]. All these techniques have high and low affinity for hydrogen and poisonous gases, respectively, but their limitations are not avoidable. For instance, the microencapsulation technique, which involves coating of the bulk alloy with 10 wt.% of Ni or Cu, utilises large amounts of the coating metal that is not responsible for hydrogen storage. It is also not economically favoured and produces heavy metal alloys [10]. On the other hand, surface modification through deposition of platinum group metals (PGMs), particularly palladium (Pd) which has a strong affinity for hydrogen, has been reported to possess relatively favourable and efficient improvement towards hydrogenation properties of alloy materials [13,14]. The effect of Pd on hydrogenation properties of alloy materials has been studied intensively over the years. For example, a study by Zaluska et al. [15] showed that, to some extent, Pd coating on AB, A_2_B, and AB_5_ alloy materials promoted fast hydrogen absorption, with a small or no incubation period. Similar observations were shared by Uchida et al. [16] when Pd nanoparticles were deposited on the surface of titanium films. In this investigation, autocatalytic palladium deposition was identified for surface modification of TiMn_1.52_ alloy; its effect on hydrogen sorption kinetics after exposure to air was then studied using a Sieverts-type apparatus. To the best of our knowledge, such studies on hydrogenation kinetics of Pd-coated TiMn_1.52_ alloy do not appear to have been reported yet.

## 2. Experimental Methods

### 2.1. Materials

The AB_2_-type (TiMn_1.52_) hydride-forming alloy was prepared from Ti (99.9%) and Mn (99.9%) purchased from Sigma Aldrich (St. Louis, MO, USA). The AB_2_ hydride forming alloy was prepared by arc-melting on a water-cooled copper crucible in a protective argon atmosphere. All prepared ingots were melted three times to provide homogeneity. Subsequently, the prepared metal ingots were pulverised by ball-milling in argon for 10 min. The material was allowed constant exposure to air throughout the experimental studies.

### 2.2. Surface Modification of Alloy

Surface modification of the TiMn_1.52_ alloy was conducted through autocatalytic deposition of Pd in a hypophosphite-based autocatalytic plating bath following the procedure described here [17]. Prior to palladium deposition, the materials were first sensitized and activated in a palladium–tin (Pd–Sn) colloidal solution [17] resulting in increased densities of Pd deposition and surface Pd loading on the intermetallide. The activated intermetallide was subsequently suspended in the palladium plating bath. An equivalent volume of NaH_2_PO_2_ solution (10 g/L) was added separately. The plating time and stirring rate were fixed at 30 min and 300 rpm, respectively. The main purpose of surface coating with Pd was to improve the poisoning-tolerance of the material as well as to form a material with excellent hydrogen sorption properties. The autocatalytic deposition of Pd was applied to a ~5 g batch of TiMn_1.52_ alloy.

### 2.3. Characterisation Techniques

X-ray diffraction (XRD) studies of the alloys were performed using a Bruker Advance powder diffractometer (Madison, WI, USA; 40 mA, 40 keV) at the Materials Research Group, iThemba Labs, in Cape Town, South Africa for phase identification. The XRD analysis was done with an X-ray source of Cu Kα radiation (λ = 1.5406 Å).

Scanning electron microscopes/energy-dispersive spectroscopy (SEM/EDS, Edax Genesis, Tilburg, The Netherlands, 100 live seconds) studies were carried out using a Leo 1450 microscope (Carl Zeiss, Jena, Germany) (20 kV, secondary electrons) at the Physics department, University of the Western Cape (UWC) to evaluate the morphology of AB_2_-type alloy particle size/shape, Pd particle dispersion on the surface of the AB_2_-type alloy particles, and Pd particle size/shape.

The effect of autocatalytic palladium deposition on hydrogenation kinetics of TiMn_1.52_ alloy was evaluated by a comparison of hydrogen absorption after pre-exposure to air and hydrogen absorption after vacuum heating at 300 °C. Vacuum heating facilitates the removal of any existing oxide layers on the surface of the alloy. Hydrogen absorption was conducted using a commercial Sieverts-type volumetric installation (PCTPro-2000, Hy-Energy LLC, California, CA, US) at the South African Institute for Advanced Material Chemistry (SAIAMC), UWC. The measurements were carried out at T = 20 °C, P_0_ ~ 30 bar H_2_, for 2 h. The experimental results were processed by application of formal kinetic analysis, using the Avrami–Erofeev Equation (1) [18]

(1) $$\left(\frac{H}{AB_{2}}\right)={\left(\frac{H}{AB_{2}}\right)}_{\max}\times \;\left\{1-\exp\left(-kt^{n}\right)\right\}$$

where *(H/AB_2_)* is the actual hydrogen concentration in the alloy, *(H/AB_2_)*_max_ is the maximum hydrogen concentration in the alloy, *t* is time, *k* represents rate constant, and the index of power, *n*, is interpreted as a value indirectly connected to the reaction mechanism.

## 3. Results and Discussion

### 3.1. Structural Characterisation

**Figure 1:**
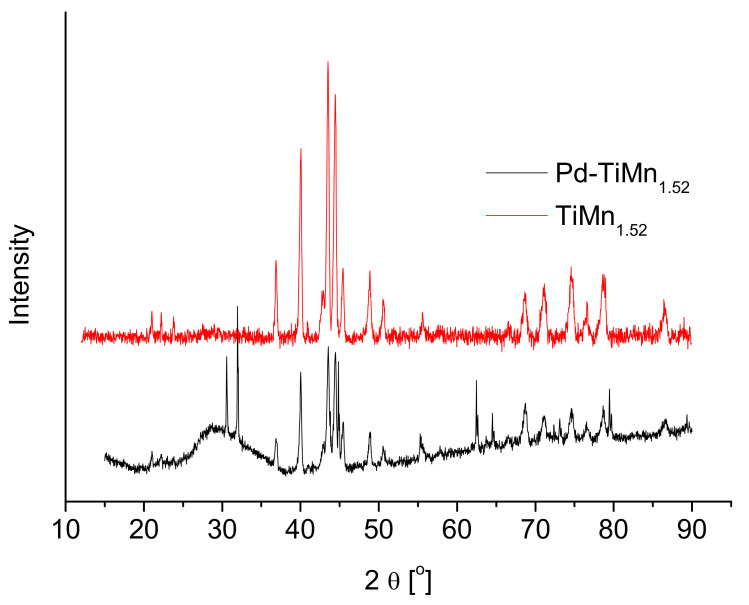
X-ray diffractograms of TiMn_1.52_ and Pd–TiMn_1.52_.

Figure 1 shows the XRD patterns of Pd-coated and uncoated TiMn_1.52_ alloys. The XRD analyses indicate that TiMn_1.52_ alloy exhibits a disordered structure and C14-type Laves phase without any second phase. C14-type Laves phase of the same alloy was previously reported by Dekhtyarenko et al. [19] and Hu et al. [8]. The most interesting feature about C14-type Laves hydrogen storage materials is that they have favourable hydrogen absorption/desorption kinetics, exhibiting easy penetration of hydrogen atoms [20]. For Pd-coated alloy, two sharp diffraction peaks of much higher intensities than those of TiMn_1.52_ alloy appear at 2θ = 30.56° and 2θ = 31.83°. The peaks are attributed to (021) and (040) reflections of phosphorus structure, respectively [21]. The phosphorus was impregnated into the Pd layer during plating of the NaH_2_PO_2_-derived Pd layer. In addition, another two peaks appeared at 2θ = 62.49° and 2θ = 64.82°. Crystallite sizes of the two alloys were calculated using Scherrer’s equation (Equation (2)) [22], where the peak at 2θ = 40.05° was used as a representative peak.

(2) $$\tau =\left(\kappa \lambda \right)∕\beta \cos\left(\theta \right)$$

Both the uncoated and Pd-coated alloys were found to have the same crystallite size of 29 nm, suggesting that there was no admixing/incorporation between palladium nanoparticles and the bulk TiMn_1.52_ alloy.

### 3.2. Morphological and Elemental Characterisations

**Figure 2:**
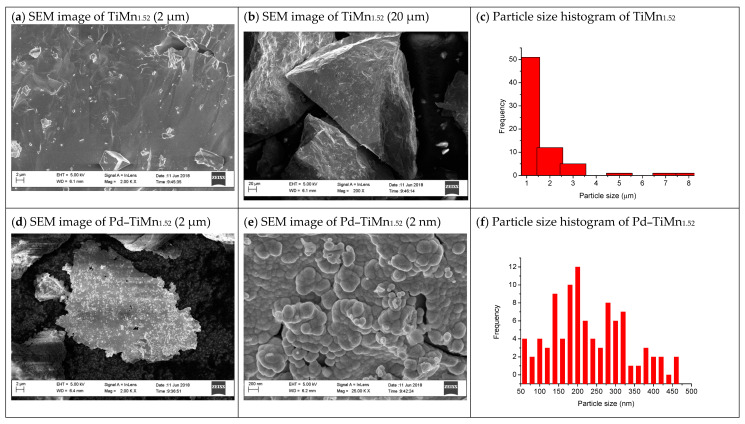
Scanning electron microscope (SEM) micrographs of (**a**,**b**) TiMn_1.52_ and (**d**,**e**) Pd–TiMn_1.52_ and their particle size distribution histograms ((**c**) TiMn_1.52_ and (**f**) Pd–TiMn_1.52_).

Figure 2 presents SEM images of TiMn_1.52_ before and after surface coating by autocatalytic palladium deposition. The uncoated alloy exhibited a relatively smooth surface, which was occupied by irregular-shaped particles varying in size from 1 to 8 µm. A particle size distribution histogram of the material indicated that the majority of the particles had a size of 1 µm. The alloy may be classified as a nonporous material. For the sample coated with Pd, a discontinuous layer of near-spherical Pd particles was observed. Moreover, the layer seemed to be very dense and uniform. A particle size distribution histogram (Figure 2f) estimated that this alloy had particles ranging in size from 50 to 475 nm, while the majority of the particles had a particle size of 200 nm.

**Figure 3:**
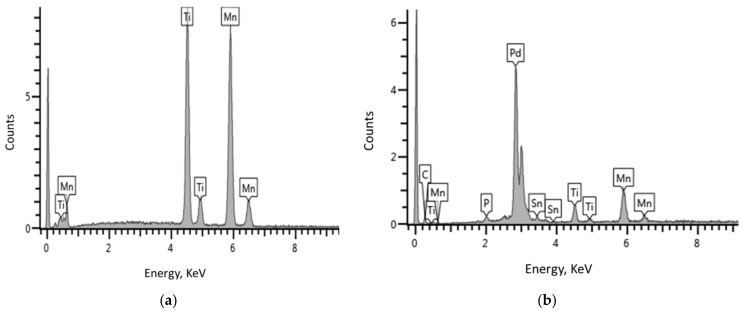
Energy dispersive spectroscopy (EDS) plots of (**a**) TiMn_1.52_ and (**b**) Pd–TiMn_1.52_.

EDS analyses (Figure 3) were employed in parallel to the SEM studies in order to determine the elemental compositions of the alloys. Table 1 show that EDS data correspond very well with the targeted composition of TiMn_1.52_ alloy, indicating a successful admixing of Ti and Mn metals (ratio of 1:1.52) through the arc melting process.

The EDS of Pd-coated alloy (Figure 3b) reveals that the impurities are phosphorus, carbon, and tin at a level of 0.70, 2.31, and 4.54 wt.%, respectively. The presence of these impurities may have resulted from palladium–tin (Pd–Sn) colloidal solution during sensitisation and activation of AB_2_ alloys as well as from the NaH_2_PO_2_-based plating bath during autocatalytic deposition of palladium. When comparing the EDS graphs (Figure 3a,b) of the two alloys, we observe that the net counts of Ti and Mn decreased from 10.25 and 9.7 to 0.9 and 1.5, respectively. This observation may be attributed to a successful palladium loading that covered most part of the alloy surface with a net count of 4.8, as witnessed in the EDS data.

### 3.3. Hydrogen Absorption Kinetics

**Figure 4:**
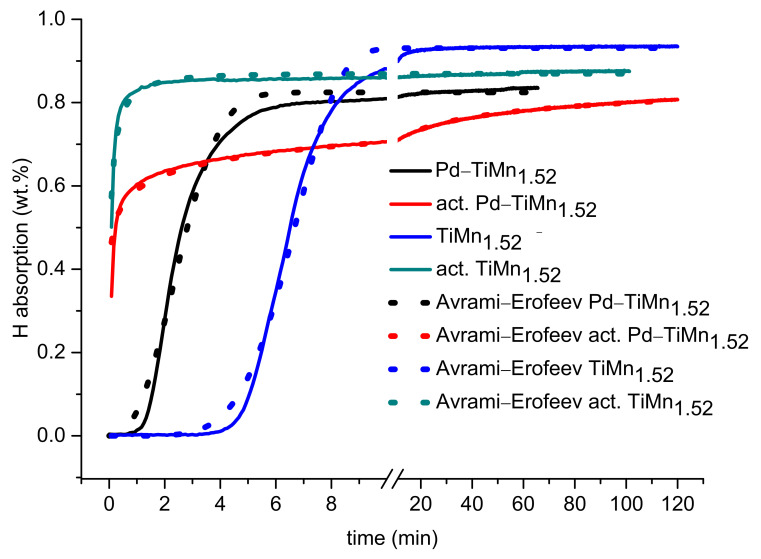
Dynamics of hydrogen absorption (T = 20 °C, P_H2_ = 30 bar) by uncoated and Pd-coated AB_2_ alloys after pre-exposure to air and after preliminary activation (vacuum heating).

Studies of the hydrogenation performances of both uncoated and Pd-coated TiMn_1.52_ alloy were conducted after pre-exposure to air and after preactivation by vacuum heating. The hydrogenation kinetic curves are presented in Figure 4. In addition, Table 2 presents the results obtained through fitting of the experimental data on the Avrami–Erofeev model, which is described here [17]. Without vacuum activation, the uncoated TiMn_1.52_ alloy exhibits slow hydrogen absorption, accompanied by a long incubation period of ~5 min. This is attributed to the presence of a poisonous oxide film on the surface, which causes difficulties in transportation of atomic hydrogen into the bulk alloy. An index of power of 1.37 (Table 2) signifies that hydrogen absorption for unmodified TiMn_1.52_ alloy without activation is controlled by nucleation and growth mechanisms.

Upon activation in a vacuum by heating at 300 °C for 2 h, the hydrogenation kinetics of TiMn_1.52_ alloy significantly improved and this was supported by the sudden increase of rate constant from 0.438 to 13.2 min^−1^ (Table 2). This is due to the fact that vacuum heating results in the removal of any oxide layers, producing a fast hydrogen-absorbing surface. However, the maximum hydrogen absorption capacity for the activated uncoated alloy was found to be lower than that for the nonactivated uncoated alloy, as depicted in Table 2. Subsequently, the maximum hydrogen absorption capacity continued to drop after electroless Pd coating. A similar trend was observed by Davids et al. [23] when loading Pd on a TiFe alloy surface.

Loss in hydrogen absorption capacity after Pd coating may be attributed to a large metal (Pd) loading on the surface of TiMn_1.52_ alloy. Li et al. [24] recommended that in order to avoid losses in hydrogenation behaviour, the total weight of PGMs during surface modification of metal hydride-forming alloy through PGMs should be in trace amounts (≤0.1 wt.%). In our case, EDS analysis revealed more than 0.1 wt.% of Pd particles on the surface of TiMn_1.52_ alloy, and a decrease in maximum hydrogen absorption capacity was observed upon Pd deposition. In addition, the presence of impurities such as Sn and C on the surface of Pd-coated alloy might have hindered complete hydrogen absorption by the material. Although a decrease in hydrogen capacity was observed, there was an improvement in hydrogenation kinetics, carried out without activation by vacuum heating, upon Pd-coating. The enhancement can be attributed to the partial removal of surface oxide films upon autocatalytic deposition of palladium as well as to the catalytic activity of Pd(P) nanoparticles facilitating splitting of hydrogen molecules into hydrogen atoms. The incubation period is shorter for the alloy coated with Pd.

After vacuum heating, the surface coated alloy exhibited faster hydrogen absorption performances without the presence of an incubation period as compared to the surface coated alloy without vacuum heating. Its hydrogenation kinetics are slower than for activated unmodified TiMn_1.52_ alloy. This is supported by a higher rate constant of activated unmodified alloy (k = 13.2 min^−1^) as compared to that of activated modified alloy (k = 1.05 min^−1^). The activated uncoated alloy together with inactivated and activated Pd-coated alloys all exhibited an index of power of between 0.5 and 1. Therefore, their interaction with hydrogen is controlled by the nucleation mechanism.

## 4. Conclusions

This study presents the effect of autocatalytic deposition of palladium on the structure, morphology, and hydrogenation kinetics of TiMn_1.52_ alloy. The study demonstrates that surface modification of TiMn_1.52_ alloy through Pd deposition results in the formation of a discontinuous layer of Pd nanoparticles on the surface of the alloy, thus causing relatively improved activation performances and hydrogen absorption kinetics even after exposure to air. The effect was attributed to improved H_2_ dissociation on Pd nanoparticles. The maximum hydrogen absorption capacity of the material decreased upon Pd deposition, and this was associated with a large metal loading on the surface.

## Figures and Tables

**Table 1:** Elemental composition data of TiMn_1.52_ alloy.

Material	Element	EDS Data		Target Composition
		Net Counts	Wt.%	Wt.%
**TiMn_1.52_**	Ti	10.25	37.00	36.44
	Mn	9.7	63.00	63.56
	Total	-	100	100

**Table 2:** Fit of the experimental data for hydrogen absorption by nonactivated and activated materials using the Avrami–Erofeev equation.

Material	${\left(\frac{H}{AB2}\right)}_{\max}\;(\mathrm{wt}.\%)$		Rate Constant, k (min^−1^)		Index of Power, n	
**State**	Non-activated	Activated	Non-activated	Activated	Non-activated	Activated
**TiMn_1.52_**	0.933	0.869	0.438	1.05	1.37	0.50
**Pd–TiMn_1.52_**	0.827	0.779	0.771	13.2	0.925	0.50

## Data Availability

Data is contained within the article.
